# Acute Respiratory Failure due to Pulmonary Toxoplasmosis in an Immunocompetent Patient: A Case Report

**DOI:** 10.1155/crcc/7875906

**Published:** 2026-09-02

**Authors:** María Jesús Ojeda, María Isabel Domínguez, Leonardo Soto, Rodrigo Kemeny, Pablo Regueira, Guillermo Ávila, Tomas Regueira

**Affiliations:** ^1^ Critical Care Unit, Clínica Universidad de los Andes, Santiago, Chile; ^2^ Department of Infectious Diseases, Clínica Santa María, Santiago, Chile, clinicasantamaria.cl; ^3^ Faculty of Medicine, Universidad de los Andes, Santiago, Chile, uniandes.edu.co; ^4^ Faculty of Medicine, Universidad del Desarrollo, Santiago, Chile, udd.cl; ^5^ Department of Internal Medicine, Clínica Santa María, Santiago, Chile, clinicasantamaria.cl

**Keywords:** acute respiratory failure, critical care, immunocompetent host, mechanical ventilation, polymerase chain reaction, pulmonary toxoplasmosis, *Toxoplasma gondii*

## Abstract

Pulmonary toxoplasmosis is a rare but potentially life‐threatening manifestation of *Toxoplasma gondii* infection in immunocompetent individuals. Clinical presentation is often nonspecific and may mimic viral or atypical pneumonia, leading to delayed diagnosis. We report the case of a previously healthy 32‐year‐old man who presented with a mononucleosis‐like syndrome complicated by rapidly progressive hypoxemic respiratory failure requiring invasive mechanical ventilation. Initial evaluation revealed marked systemic inflammation, cytopenias, hyperferritinemia, and mixed‐pattern hepatitis. Chest computed tomography demonstrated bilateral consolidations, ground‐glass opacities, pleural effusions, and interstitial changes. Extensive microbiological and immunological investigations were initially unrevealing. Polymerase chain reaction (PCR) testing for *T. gondii* was positive in blood despite negative IgG serology and isolated IgM positivity, raising concern for potentially misleading serological findings. The diagnosis of pulmonary toxoplasmosis was subsequently confirmed by PCR detection of *T. gondii* DNA in bronchoalveolar lavage fluid. Targeted treatment with trimethoprim–sulfamethoxazole led to rapid clinical and biochemical improvement, allowing successful extubation after 6 days of mechanical ventilation and complete radiological resolution prior to discharge. This case highlights the diagnostic challenges of pulmonary toxoplasmosis in immunocompetent hosts and underscores the importance of molecular testing in critically ill patients with severe pneumonia and ambiguous serological findings.

## 1. Introduction

*Toxoplasma gondii* is an obligate intracellular protozoan parasite with a worldwide distribution, infecting nearly one‐third of the global population [1]. In immunocompetent individuals, infection is most often asymptomatic or manifests as a mild, self‐limited illness. Nevertheless, the parasite has the capacity to establish lifelong latency and may reactivate in the setting of impaired host immunity^1^. Human infection occurs predominantly through ingestion of oocysts shed in feline feces, consumption of tissue cysts in undercooked meat, or via vertical transmission during pregnancy^2^. Despite its high prevalence, toxoplasmosis is frequently overlooked as a cause of systemic infection in adults, largely because of its nonspecific clinical presentation [2].

In immunocompetent hosts, acute toxoplasmosis typically presents as a mononucleosis‐like syndrome, characterized by fever, malaise, lymphadenopathy, and mild elevations in liver transaminases [2]. The clinical course is generally benign and self‐limited; however, atypical or severe organ involvement may occasionally occur, leading to diagnostic uncertainty and misclassification as viral or inflammatory disease [2]. Serological testing remains the cornerstone of diagnosis, with detection of IgM and IgG antibodies providing evidence of exposure. Interpretation of serology can be challenging, as IgM antibodies may persist for several months after acute infection and may yield false‐positive results. In recent years, molecular techniques such as polymerase chain reaction (PCR) have become increasingly valuable, particularly in severe or disseminated presentations, allowing direct detection of *T. gondii* DNA and improving diagnostic accuracy [2].

Pulmonary involvement in toxoplasmosis is uncommon, particularly in immunocompetent individuals, but when present it may result in severe clinical manifestations, including hypoxemic respiratory failure and acute respiratory distress syndrome [3–5]. Classically, *T. gondii* pneumonia has been described in patients with advanced HIV infection, hematologic malignancies, or following organ transplantation; however, an increasing number of cases have been reported in previously healthy adults [4, 5]. Radiologic findings are nonspecific and often include bilateral ground‐glass opacities or interstitial infiltrates, which overlap with more frequent causes of severe pneumonia. As a result, pulmonary toxoplasmosis is rarely considered early in the diagnostic workup, potentially delaying appropriate treatment [4].

Here we present a case of a previously healthy young adult with a mononucleosis‐like illness complicated by severe pulmonary toxoplasmosis requiring mechanical ventilation.

## 2. Case

We report the case of a 32‐year‐old previously healthy man who initially developed progressive asthenia and reduced exercise tolerance. This was followed by odynophagia, for which he self‐medicated with oral prednisone at a dose of 20 mg every 12 h for 2–3 days, achieving only partial symptomatic relief. Four days later, he developed persistent high‐grade fever, with temperatures reaching 39.1°C, accompanied by myalgias, anorexia, abdominal distension, and a single episode of emesis.

Six days after symptom onset, the patient presented to the emergency department. Initial laboratory evaluation revealed a leukocyte count of 5100/mm^3^ with 12% band forms and an elevated C‐reactive protein (CRP) level of 33 mg/L. He was discharged with symptomatic management. However, he represented within 24 h due to worsening general condition and the development of respiratory symptoms, including dyspnea. Repeat laboratory testing demonstrated marked hematologic deterioration, with severe leukopenia (1900/mm^3^) and a pronounced left shift (25% band forms, 31% segmented neutrophils), thrombocytopenia (88,000/mm^3^), and further elevation of CRP (65 mg/L). Biochemical abnormalities included elevated transaminases, lactate dehydrogenase (LDH) of 603 U/L, alkaline phosphatase of 140 U/L, and total bilirubin of 2.01 mg/dL.

**Figure 1:**
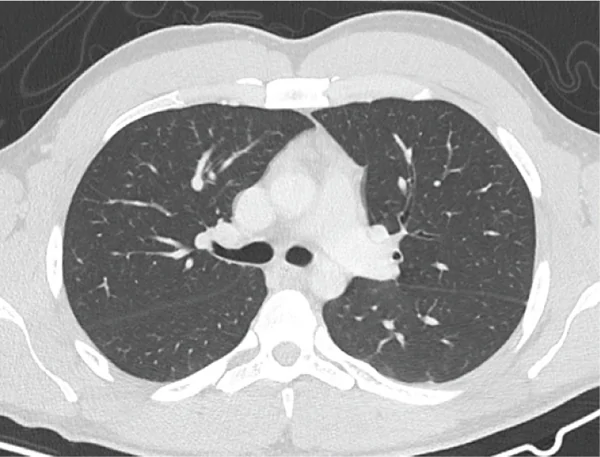
Initial chest computed tomography demonstrating normal lung parenchyma, without consolidations, ground‐glass opacities, or pleural effusion.

Computed tomography of the chest (Figure 1), abdomen, and pelvis demonstrated small mesenteric lymphadenopathies with associated panniculitis, nonspecific enlargement of left axillary lymph nodes, and mild splenomegaly. The patient was admitted to the intermediate care unit with a working diagnosis of fever of unknown origin. An extensive etiologic evaluation was initiated and was negative for hepatitis B and C viruses, HIV infection, bacterial meningitis by PCR, cytomegalovirus (IgM/IgG negative), Epstein–Barr virus (IgM negative, IgG positive), *Treponema pallidum* (VDRL), hepatitis A virus IgM, *Bartonella henselae* IgM/IgG, and HIV viral load. Transthoracic echocardiography showed no evidence of infective endocarditis.

After 3 days of hospitalization in intermediate care with stable clinical status, the patient was transferred to the general ward. However, later that night he experienced abrupt clinical deterioration, characterized by worsening dyspnea, increased work of breathing, and persistent high‐grade fever unresponsive to antipyretic therapy. Repeat laboratory testing revealed a marked hyperinflammatory profile, including ferritin levels exceeding 5000 ng/mL, triglycerides > 150 mg/dL, fibrinogen 422 mg/dL, and a further rise in CRP to 106 mg/L. Liver enzymes were markedly elevated (AST 596 U/L, ALT 890 U/L), accompanied by elevated LDH (633 U/L), hypoalbuminemia (3.15 g/dL), and mild renal dysfunction (creatinine 1.23 mg/dL).

**Figure 2:**
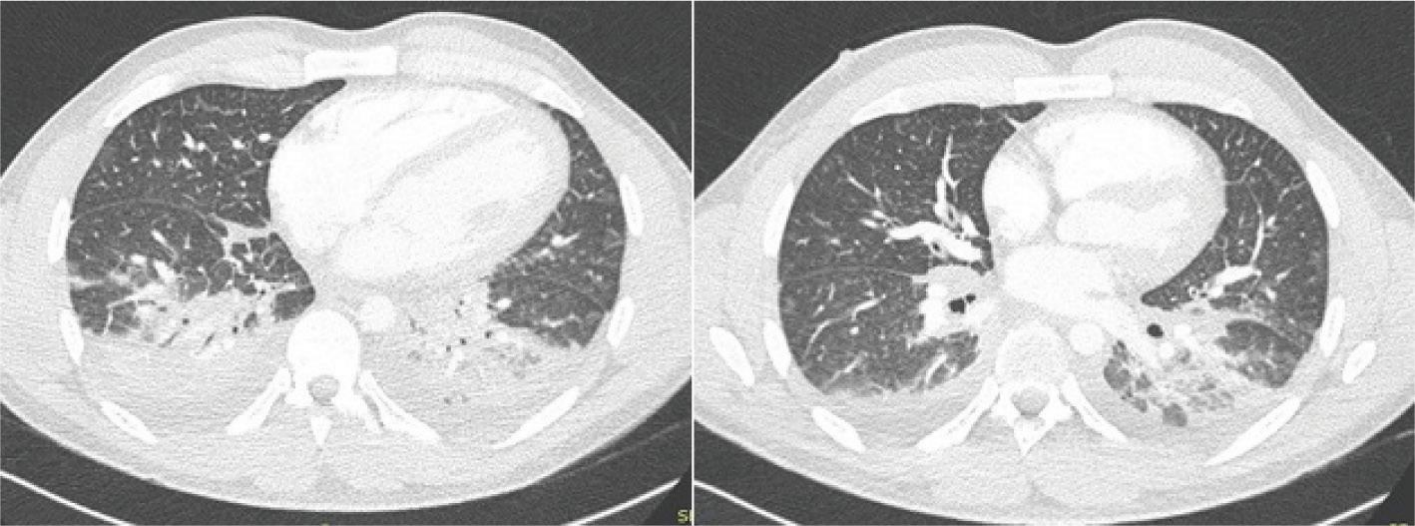
Chest computed tomography pulmonary angiography excluding pulmonary embolism and demonstrating bilateral bibasilar consolidations with areas of alveolar filling predominantly in the lower lobes, associated inflammatory airway changes, moderate bilateral pleural effusions, and features of interstitial pulmonary edema.

Chest computed tomography pulmonary angiography excluded pulmonary embolism but demonstrated bilateral bibasilar consolidations with areas of alveolar filling predominantly in the lower lobes, inflammatory airway changes, moderate bilateral pleural effusions, and signs of interstitial pulmonary edema (Figure 2). Repeat transthoracic echocardiography remained within normal limits. In the setting of increasing oxygen requirements, the patient was transferred back to the intermediate care unit, and empiric antimicrobial therapy with ceftriaxone and azithromycin was initiated. Over the following 24 h, the patient developed progressive hypoxemia requiring escalation to high‐flow nasal cannula and subsequent transfer to the intensive care unit. He was intubated for worsening respiratory distress and severe hypoxemia, with a PaO_2_/FiO_2_ ratio of 90.

An extensive diagnostic workup was undertaken, including bronchoscopy with bronchoalveolar lavage (BAL), which revealed marked cellularity (26.5 × 10^6^ white blood cells/100 mL) with a significant lymphocytic predominance (73%), macrophages accounting for 25%, and no eosinophils. Gram stain and bacterial cultures from BAL were negative. Immunological evaluation demonstrated a CD4 count at the lower limit of normal with relative CD4 lymphopenia, whereas CD3, CD8, CD16/56, and B‐lymphocyte populations were within normal ranges. Soluble CD25 levels were elevated (2900 pg/mL). Given the combination of fever, cytopenias, splenomegaly, hyperferritinemia and elevated soluble CD25, a secondary hemophagocytic (HLH) syndrome was actively considered. The patient fulfilled four of the eight HLH‐2004 criteria and therefore did not meet the diagnostic threshold of ≥ 5 criteria; notably, fibrinogen was elevated (422 mg/dL) rather than reduced, and triglycerides did not reach the diagnostic threshold. Therefore, the observed hyperinflammatory profile was interpreted as a secondary, infection‐driven inflammatory response rather than as established HLH. Accordingly, bone marrow aspiration and NK‐cell functional testing were not performed, although quantitative CD16/56+ NK‐cell numbers, as pointed before, were normal. Additionally, systemic lupus erythematosus and ANCA‐associated vasculitis were excluded.

A probable etiologic diagnosis was established when PCR testing for *T. gondii* in blood returned positive, in the context of negative anti‐*Toxoplasma* IgG antibodies (0.01 IU/mL) and positive IgM antibodies (7.17 IU/mL; positive > 0.65). Targeted antimicrobial therapy with trimethoprim–sulfamethoxazole (TMP‐SMX) was promptly initiated, and empiric ceftriaxone and azithromycin were discontinued. The diagnosis of pulmonary toxoplasmosis in an immunocompetent patient was subsequently confirmed by a positive PCR for *T. gondii* in BAL fluid.

After diagnosis, a more comprehensive assessment of host immune status was undertaken to exclude an underlying or secondary immunodeficiency. The patient had no personal or family history of recurrent, severe or opportunistic infections, and no history of malignancy, autoimmune disease, solid‐organ or hematopoietic transplantation, splenectomy, diabetes mellitus, chronic liver or kidney disease, or malnutrition. He was not receiving immunosuppressive, cytotoxic or biologic therapy. HIV infection was excluded by negative serology together with an undetectable HIV‐1 viral load, thereby also excluding acute retroviral syndrome within the serological window period. Quantitative serum immunoglobulins were within normal limits.

The patient showed rapid clinical improvement, with progressive declines in ferritin, LDH, and inflammatory markers over the following 48–72 h. He was successfully extubated 6 days after initiation of mechanical ventilation, with resolution of acute kidney injury and gradual improvement of mixed‐pattern transaminitis. A follow‐up chest CT performed prior to discharge demonstrated near‐complete resolution of pulmonary infiltrates and pleural effusions.

During postintensive care follow‐up, repeat serological testing demonstrated seroconversion of anti‐*T. gondii* IgG antibodies, with IgG levels increasing to 44.0 IU/mL, confirming recent primary infection. This delayed IgG seroconversion supports the diagnosis of acute toxoplasmosis.

## 3. Discussion

Severe pulmonary toxoplasmosis in immunocompetent individuals is rare, but reported cases describe a remarkably consistent clinical and radiologic pattern. Typical findings include bilateral ground‐glass opacities, periseptal and peribronchovascular thickening, pleural effusions, progressive hypoxemia, and rapid evolution to acute respiratory failure, often mimicking other causes of atypical pneumonia [4, 6]. Several case series report that up to one‐third of immunocompetent patients require invasive mechanical ventilation, frequently accompanied by marked systemic inflammation and a lymphocytic predominance in BAL fluid [5]. Given the nonspecific nature of both imaging and clinical presentation, pulmonary toxoplasmosis is unlikely to be suspected unless *T. gondii* is considered early in patients presenting with severe pneumonia following a mononucleosis‐like prodrome [2].

The diagnosis of pulmonary toxoplasmosis requires integration of clinical, radiologic, and microbiological findings, with molecular detection of *T. gondii* DNA in blood or BAL fluid playing a central role [4, 5]. Early recognition is critical, as prompt initiation of effective antimicrobial therapy—most commonly TMP‐SMX or pyrimethamine‐based regimens—is associated with rapid clinical improvement and favorable outcomes [1]. A major diagnostic pitfall in acute toxoplasmosis is the interpretation of serology, particularly false‐positive IgM results. IgM antibodies may persist for months after infection, cross‐react with other pathogens, or reflect nonspecific polyclonal B‐cell activation during systemic inflammatory states [7]. Consequently, isolated IgM positivity should not be regarded as definitive evidence of acute infection in immunocompetent adults. In this setting, PCR‐based detection of *T. gondii* in blood or BAL fluid becomes essential to confirm active disease, especially in critically ill patients [2]. In addition, many patients exhibit pronounced inflammatory responses—including elevated CRP, hyperferritinemia, cytopenias, and biochemical features of mild hepatitis—which overlap with viral infections such as EBV or CMV or hematological syndromes and further complicate early diagnostic assessment [8]. In a young adult, these findings direct the diagnostic effort toward a hematologic or hyperinflammatory disorder—lymphoma, acute leukemia, HLH, or systemic autoimmune disease—rather than toward an infectious cause, and this misdirection is itself an important source of diagnostic delay. In our patient, the initial working diagnosis was fever of unknown origin, and the extensive hematologic and immunologic workup that ensued preceded, rather than prompted, the molecular testing that ultimately established the etiology. We would therefore emphasize that in this clinical picture the search for a treatable infectious trigger—including molecular testing for *T. gondii*—should proceed in parallel with, and not after, the evaluation for hyperinflammatory and hematologic syndromes. In our case, the diagnosis was further supported by delayed IgG seroconversion documented during post‐ICU follow‐up, reinforcing the limitations of early serology and the diagnostic value of molecular testing in the acute phase.

Furthermore, the radiological evolution in our patient deserves comment. The chest CT performed on admission, 6 days after symptom onset, demonstrated normal lung parenchyma, without consolidation, ground‐glass opacity or pleural effusion (Figure 1). Within approximately 72 h, and concurrently with the abrupt clinical deterioration and the rise in ferritin, LDH and CRP, repeat imaging showed bilateral bibasilar consolidations, alveolar filling, moderate bilateral pleural effusions, and interstitial changes (Figure 2), followed by near‐complete resolution before discharge. This sequence differs from the more indolent course of most atypical pneumonias and is consistent with rapid hematogenous dissemination of the parasite to the lung. We suggest that, in a patient with a mononucleosis‐like prodrome and a hyperinflammatory profile, such a fulminant radiological trajectory should raise suspicion of severe acquired toxoplasmosis and prompt early molecular testing rather than a further period of observation.

Although the combination of pyrimethamine–sulfadiazine with leucovorin has traditionally been considered the standard treatment for toxoplasmosis, accumulating evidence supports the use of high‐dose TMP‐SMX in both immunocompromised and immunocompetent patients [3]. TMP‐SMX offers several practical advantages, including widespread availability, lower cost, and good tolerability, while maintaining comparable efficacy. Published case reports describe rapid improvement in oxygenation and radiological findings following initiation of therapy [3], with treatment durations typically ranging from 14 to 21 days, tailored to clinical response. Consistent with these observations, our patient showed prompt clinical stabilization and was successfully extubated shortly after targeted therapy was initiated.

The prognosis of pulmonary toxoplasmosis in immunocompetent adults is generally favorable when the diagnosis is established early and appropriate antimicrobial therapy is promptly initiated [5]. Most patients achieve full clinical recovery, even in cases requiring temporary invasive mechanical ventilation, and long‐term pulmonary sequelae appear to be uncommon [5]. This favorable outcome is also reflected radiologically; published case series have shown complete resolution of pulmonary infiltrates, ground‐glass opacities, and pleural effusions on follow‐up computed tomography in all adequately treated immunocompetent patients [9]. In contrast, delayed recognition of the disease may lead to progression to acute respiratory distress syndrome or multiorgan involvement, with a consequent and significant worsening of clinical outcomes [4].

A further aspect of this case that merits comment is the management of respiratory support during the phase of rapid clinical deterioration. Our patient progressed from conventional oxygen to high‐flow nasal oxygen and then to invasive mechanical ventilation within less than 24 h. In de novo acute hypoxemic respiratory failure, noninvasive respiratory support carries a substantial risk of failure, and prolonging it in the presence of an intense respiratory drive may be harmful: vigorous inspiratory effort generates large tidal volumes and injurious transpulmonary pressure swings that can amplify pre‐existing lung injury, a mechanism now recognized as patient self‐inflicted lung injury [10]. Consistent with this concept, a high‐expired tidal volume during noninvasive ventilation has been shown to be independently associated with treatment failure, with values above approximately 9.5 mL/kg of predicted body weight accurately predicting the need for intubation in patients with moderate‐to‐severe hypoxemia [11]. Alternative approaches to quantify the risk of failure of noninvasive support include serial monitoring of the ROX index (the ratio of SpO_2_/FiO_2_ to respiratory rate), a validated bedside tool to identify patients at high risk of high‐flow nasal oxygen failure and thereby avoid delayed intubation [12], and the direct measurement of tidal volume and inspiratory effort using specific technical solutions [13]. In our patient, tidal volume and esophageal pressure were not monitored during the noninvasive phase, and the decision to intubate was based on conventional clinical and gasometric criteria. We nevertheless consider that the short duration of noninvasive support and the prompt escalation to invasive ventilation, followed by early bronchoscopy that ultimately yielded the diagnosis, were important contributors to the favorable outcome.

This report has limitations. Complement pathway evaluation, functional T‐cell assays (lymphocyte proliferation and IFN‐*γ* production) and genetic testing for inborn errors of immunity were not performed, so a subtle or partial immune defect cannot be entirely excluded. In addition, although the brief self‐administered course of prednisone taken during the prodromal phase, shortly before clinical deterioration, was well below the dose and duration conventionally associated with clinically significant immunosuppression, it cannot be excluded as a contributing factor to the severity of the presentation.

## 4. Conclusion

This case underscores the need to consider *T. gondii* infection in patients presenting with severe pneumonia following a mononucleosis‐like syndrome, particularly when serological patterns are ambiguous and inflammatory biomarkers broaden the differential diagnosis. Early molecular confirmation and timely initiation of therapy remain critical determinants of a good outcome.

## Funding

No funding was received for this manuscript.

## Disclosure

The authors reviewed and edited the content as needed and take full responsibility for the content of the publication.

## Consent

Written informed consent was obtained from the patient for publication of this case and any accompanying images or clinical information.

## Conflicts of Interest

The authors declare no conflicts of interest.

## Data Availability

The data that support the findings of this study are available on request from the corresponding author. The data are not publicly available due to privacy or ethical restrictions.
